# The Central Nervous System Contains ILC1s That Differ From NK Cells in the Response to Inflammation

**DOI:** 10.3389/fimmu.2019.02337

**Published:** 2019-10-10

**Authors:** Silvina Romero-Suárez, Alba Del Rio Serrato, Roemel Jeusep Bueno, Daniel Brunotte-Strecker, Christina Stehle, Caio Andreeta Figueiredo, Laura Hertwig, Ildiko R. Dunay, Chiara Romagnani, Carmen Infante-Duarte

**Affiliations:** ^1^Charité - Universitätsmedizin Berlin, Corporate Member of Freie Universität Berlin, Humboldt-Universität zu Berlin, Berlin Institute of Health, Institute for Medical Immunology, Berlin, Germany; ^2^Innate Immunity, German Rheumatism Research Center (DRFZ), Leibniz Association, Berlin, Germany; ^3^Medical Faculty, Institute of Inflammation and Neurodegeneration, Otto von Guericke University Magdeburg, Magdeburg, Germany; ^4^Department of Medicine Huddinge, Center for Infectious Medicine, Karolinska Institutet, Karolinska University Hospital, Stockholm, Sweden

**Keywords:** innate lymphoid cells (ILCs), ILC1s, natural killer (NK) cells, central nervous system (CNS), experimental autoimmune encephalomyelitis (EAE), mouse

## Abstract

Innate lymphoid cells (ILCs) are tissue resident cells with organ-specific properties. Here, we show that the central nervous system (CNS) encompasses ILCs. In particular, CD3^−^NK1.1^+^ cells present in the murine CNS comprise natural killer (NK) cells, ILC1s, intermediate ILC1s (intILC1s) and ex-ILC3s. We investigated the properties of CNS-ILC1s in comparison with CNS-NK cells during steady state and experimental autoimmune encephalomyelitis (EAE). ILC1s characteristically express CXCR3, CXCR6, DNAM-1, TRAIL, and CD200R and display heightened TNF-α production upon stimulation. In addition, ILC1s express perforin and are able to degranulate, although in a lesser extent than NK cells. Within the CNS compartments, ILC1s are enriched in the choroid plexus where very few NK cells are present, and also reside in the brain parenchyma and meninges. During EAE, ILC1s maintain stable IFN-γ and TNF-α levels while in NK cells the production of these cytokines increases as EAE progresses. Moreover, the amount of ILC1s and intILC1s increase in the parenchyma during EAE, but in contrast to NK cells, they show no signs of local proliferation. The upregulation in the inflamed brain of chemokines involved in ILC1 migration, such as CXCL9, CXCL10, and CXCL16 may lead to a recruitment of ILC1s from meninges or choroid plexus into the brain parenchyma. In sum, CNS-ILC1 phenotype, distribution and moderate inflammatory response during EAE suggest that they may act as gatekeepers involved in the control of neuroinflammation.

## Introduction

Innate lymphoid cells (ILCs) are tissue resident cells (1) that contribute to tissue homeostasis and react early to local inflammatory events [reviewed in (2)]. The long-known conventional natural killer (NK) cells are now categorized as cytotoxic ILCs, which share phenotypical and functional similitudes with helper-like ILC1s (3). Although ILC1s and NK cells develop from different precursor cells (4, 5), both express the T-box family transcription factor T-bet and secrete type I cytokines, such as interferon-γ (IFN-γ). Apart from to T-bet, NK cells express and depend on the transcription factor Eomesodermin (Eomes) for their development (6, 7). Before the formal definition of ILCs (3), ILC1s and NK cells were often confounded and evaluated as a unique cell population due to their similar phenotype and function, i.e., their primary role in protecting the host against intracellular infections and cancer. Classically, NK cells were defined in the mouse as CD3-negative cells that express the natural cytotoxicity receptor (NCR) NKp46 and NK1.1 (8–10); receptors that are also expressed on ILC1s and a subset of ILC3s (5, 11). However, a particular expression pattern of integrins can aid in the distinction between these two ILC types. While CD3^−^NK1.1^+^/NKp46^+^ circulating or splenic NK cells express the α2 integrin CD49b (DX5 antigen) (12), ILC1s express the α1 or αE forms (CD49a and CD103, respectively), and the lectin CD69, which contribute to cell retention into the tissues (13–15).

Although, in the last years, the phenotype and role of the ILC subsets in different organs have been intensively investigated, very little is known about the presence of ILCs in the central nervous system (CNS) or about their implication in neuroinflammatory diseases, such as multiple sclerosis (MS). In contrast, the role of NK cells has been widely studied in MS patients and its animal model, the experimental autoimmune encephalomyelitis (EAE). We and others have shown evidence for deficient NK cell activity in patients with MS, suggesting that NK cells may have a protective, disease-limiting role in neuroinflammation (16–19). In the EAE model, NK cells can have both detrimental (20, 21) and beneficial (22–24) roles depending on the NK cell maturation stage and disease stage investigated. In this line, we have recently shown that the mature CD11b^+^ NK cell subset is more cytotoxic toward autoreactive T cells than the immature CD11b^−^ NK cell subset and that the chemokine receptor CX3CR1 mediates the migration of mature NK cells into the CNS, contributing to the control of neuroinflammation during EAE (25). Additionally, we observed that a proportion of immature NK cells is present in the brain parenchyma in steady state and during neuroinflammation; however, whether this immature population is recruited from the periphery or whether they represent a CNS-resident ILC subset remains unclear. We hypothesized that *bona fide* ILC1s might be concealed among the phenotypically “immature NK cells.” Therefore, in the present study, we aimed to characterize the innate NK1.1^+^ (NKp46^+^) cells of the CNS.

We show that the NK1.1^+^ cells present in the healthy murine CNS include ILC1s, intILC1s and NK cells but not ILC3s. We characterized the phenotypic profile of ILC1s in comparison with NK cells identifying key ILC1 markers and investigated their presence in the different CNS compartments. In addition, we analyzed the dynamics of the different group 1 ILCs during neuroinflammation using the EAE model. The particular phenotype and dynamics of CNS-ILC1s at steady state and inflammation highlight their potential function as neuroprotective, gatekeeper and anti-inflammatory agents, opening new avenues for the study of the implication of ILC1s in CNS homeostasis.

## Materials and Methods

### Mice

WT female C57BL/6 mice were obtained from the Research Institute for Experimental Medicine (FEM) of the Charité (Berlin, Germany) and kept on a 12:12 h day:night cycle with *ad libitum* access to food and water. Rorc-Cre^Tg^; Rosa26R^RFP/+^ mice and RORc-GFP mice were provided by C. Romagnani. All animal experiments were approved by the regional animal study committee of Berlin (Landesamt für Gesundheit und Soziales) and performed in accordance to national and international guidelines.

### EAE Induction

Active EAE was induced in 8–12 weeks old female C57BL/6 mice as previously described (25). In brief, mice were immunized by a subcutaneous injection of 200 μl of myelin oligodendrocyte glycoprotein peptide 35–55 (MOG35-55) (Pepceuticals, Leicester, UK) emulsified in complete Freund's adjuvant (Difco Laboratories, USA) containing 800 μg Mycobacterium tuberculosis H37Ra (Difco). Pertussis toxin (200 ng, Sigma–Aldrich) was administered intraperitoneally at the day of immunization and 48 h later. Clinical symptoms were monitored daily and scored as follows: 0, no symptoms; 0.5, tail weakness; 1, lack of tail tone, 1.5, no righting reflex: 2, hind-limb weakness; 2.5, partial hind-limb paralysis; 3, total hind-limb paralysis; 3.5, ascending fore-limb paralysis. The mice were sacrificed when a score >3 was reached.

### Preparation of Single Cell Suspension for Flow Cytometry

Mice were euthanized under anesthesia and perfused with 60 ml of cold PBS. Tissue were collected on ice and immediately processed, blood was collected in tubes with 2 mM EDTA at room temperature. In brief, the CNS (brain and spinal cord) was mechanically homogenized with a syringe plug though a 70 μm cell strainer (Corning) and washed with RPMI 1640 medium (Gibco) supplemented with 10% fetal calf serum (FCS) and antibiotics. The myelin rich cell suspension was resuspended in 37% Percoll (Sigma-Aldrich) and the lymphocytes were collected from the pellet after centrifugation at 2,800 g. Single cell suspension of spleen and lymph nodes were obtained by homogenizing the tissue though a 100 μm cell strainer, in the case of spleen and blood, erythrocytes were lysed for 10 min with 0.15 M ammonium chloride and washed. Liver lymphocytes were enriched after mechanical dissociation and homogenization through a 100 μl cell strainer by centrifuging in a 37.5% Percoll solution.

The meninges and CP was removed from the same mouse after a thorough perfusion with cold PBS. In brief, the ventral side of the skull was carefully removed to expose the brain. The brain was removed and placed in a petri dish with cold PBS and the CP was removed under a dissecting microscope by opening the fourth, third and lateral ventricles, consecutively, and carefully detaching the CP with small forceps. In parallel, the dural meninges were peeled off from the interior side of the skull cap after scoring the edge of the skull 360° with Dumont # 5 forceps. Each tissue was kept in separate tubes with medium on ice. The brain was processed as stated above. The CP was dissociated by pipetting it through a 75 μm nylon mesh. The meninges were digested 20 min in 2 mg/ml DNAse (Sigma Aldrich #DN25) + 2.8 mg/ml collagenase from Clostridium histolyticum (Sigma Aldrich #C2139) and passed through a 75 μm nylon mesh following a washing step and cell staining.

### Flow Cytometry

Flow cytometry staining was performed at 4°C in PBS containing 0.5% BSA. Fc receptors were blocked by incubating 15 min with anti-mouse CD16/CD32 (clone 2.4G2, BD Biosciences), afterwards, monoclonal anti-mouse antibodies were added and incubated for 20 min. For intracellular and intranuclear staining, the FoxP3 transcription factor staining buffer set (Invitrogen) was used to fix and permeabilize the cells according to the manufacturer's instructions. The following antibodies were used: CD3-Pacific Blue (clone 500A2), CD11b-APC/Cy7 (clone M1/70), RORγt-Brilliant Violet 421 (clone Q31-378) from BD Biosciences; CD49b-PEDazzle 594 (clone DX5), DNAM-PE (clone 480.1), CXCR3-Brilliant Violet 510 (clone CXCR3-173), CCR7-Alexa Fluor 488 (clone 4B12), CCR6-PE/Cy7 (clone 29-2L17), CD127-PE (clone A7R34), CD122-biotin (clone TM-β1), TRAIL-biotin (clone N2B2), c-Kit-Brilliant Violet 605 (clone ACK2), CD103-biotin (clone 2E7), CD90.2-Alexa Fluor 700 (clone 30-H12), CX3CR1-PerCP/Cy5.5 (clone SA011F11), Tbet-Brilliant Violet 711 (clone 4B10), Eomes-PE/eFluor 610 (clone Dan11mag), CD200R-PE (clone OX-110), Granzyme B-Alexa Fluor 700 (clone QA16A02) and Perforin-PE (clone S16009A) from Biolegend; CD45-FITC (clone 30-F11), CD27-PE/Cy7 (clone LG.7F9), Ki67-PerCP/eFluor 710 (clone SolA15), CXCR6-PE (clone DANID2), IFNγ-PE (clone XMG1.2) and TNFα-PerCP/eFluor 710 (clone MP6-xt22) from eBioscience; NK1.1-APC (clone PK136), CD49a-PE/Vio 770 (clone REA493), CD69-PerCP/Vio 700 (clone HI.2F3), and Tbet-PE/Vio 615 (clone REA102) from Miltenyi Biotec. 7-AAD or the Live/Dead fixable cell death stain from Invitrogen were used for dead cell exclusion. Sample acquisition was performed in a BD Fortessa Flow Cytometer (BD Biosciences). Compensation and data analysis was performed in FlowJo (version 10.4, Treestar) software. Gating of populations were defined with fluorescence minus one (FMO) staining controls. Flow cytometry plots are shown as contour plots (5% with outliers). Overlay histograms were normalized to mode.

### *Ex vivo* Stimulation of CNS Cells

For intracellular cytokine detection, CNS single cell suspension derived from naive or EAE mice was stimulated in 96 well plates in complete medium [RPMI-1640 supplemented with 2 mM L-glutamine (Gibco), 100 U/mL penicillin (Seromed), 100 μg/mL streptomycin (Seromed), 10% FCS (Sigma–Aldrich) and 1% HEPES (Gibco)] with PMA (50 ng/ml, Sigma-Aldrich) and Ionomycin (1 μg/ml, Sigma-Aldrich) and incubated together with the cell transport inhibitor Brefeldine A (5 μg/ml, Biolegend) for 5 h at 37°C. Cells were then stained for surface markers, fixed and stained for cytokine detection in permeabilization buffer.

To test degranulation capacity, CNS single cell suspension from each individual mouse was incubated for 4 h at 37°C with PMA (5 ng/ml) and Ionomycin (500 ng/ml) or without stimulants as a control to assess basal degranulation in in complete medium in the presence of CD107a-PE (clone 1D4B; Biolegend), cells were then stained for surface markers and analyzed by flow cytometry.

### Quantitative Real-Time PCR

A posterior quarter of the brain of perfused mice was collected, snap frozen in liquid nitrogen and stored at −80°C until processing. RNA extraction was performed using a RNeasy Lipid Tissue Mini Kit (Qiagen). Reverse transcription was performed with MultiScribe Reverse Transcriptase (Thermofisher). RT-PCR was prepared using TaqMan Gene Expression Assays (Cxcl9, ID: Mm00434946_m1; Cxcl10, ID: Mm00445235_m1; Cxcl16, ID: Mm00469712_m; Hprt, ID: Mm03024075_m1) and TaqMan Fast Advanced Master Mix (Thermofisher) and analyzed in an ABI Prism 7500 Real-Time PCR System instrument. Target gene expression was normalized to the housekeeping gene Hprt. The fold change in expression relative to naive mice was calculated with the 2^−ΔΔ*Ct*^ method.

### Statistical Analysis

GraphPad Prism 8 was used for statistical analysis. Statistical difference between two independent groups was estimated with an unpaired two-tailed Student's *t*-test. Statistical difference among more than two groups was estimated with an ANOVA. A Shapiro-Wilk test was used to verify the normal distribution of the data. Values of *p* < 0.05 were considered significant (^*^p < 0.05, ^**^p < 0.01, ^***^p < 0.001). Figures show bars or dots indicating mean ± SEM. Due to the exploratory nature of the study, a power calculation was not performed to determine the sample size of each group, therefore a rule of minimum three animals to maximum eight animals per group was set for data sampling.

## Results

### A Fraction of NK1.1^+^CD27^+^ Cells Present in the CNS Are CD49a^+^CD49b^−^

**Figure 1:**
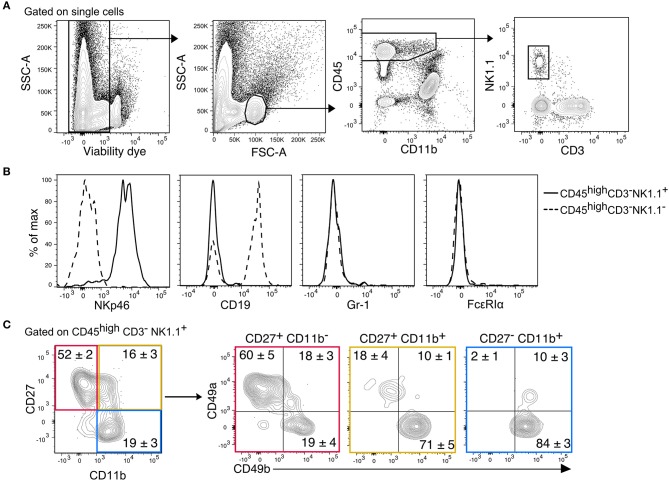
Identification of CD45^high^CD3^−^NK1.1^+^ subsets in the murine CNS. **(A)** Gating strategy to detect NK1.1^+^ cells in the CNS. After doublets and dead cell exclusion, a population that matches the size and granularity of mononuclear cells is selected, from there, the CD45^high^CD11b^−/low^ population is selected to identify the NK1.1^+^CD3^−^ cells. **(B)** Overlayed histograms of the expression of the lineage markers NKp46, CD19, Gr-1, and FcεRIα in the CD3^−^NK1.1^+^ (solid line) and CD3^−^NK1.1^−^ (dotted line) populations of the CD45^high^CD11b^−/low^ gate. Representative histograms of three mice. **(C)** Representative contour plots show the expression of CD49a and CD49b in the maturation subsets of CD3^−^NK1.1^+^ cells found in the naive CNS. The numbers represent the mean percentage ± SEM of each population (*n* = 6).

**Figure 2:**
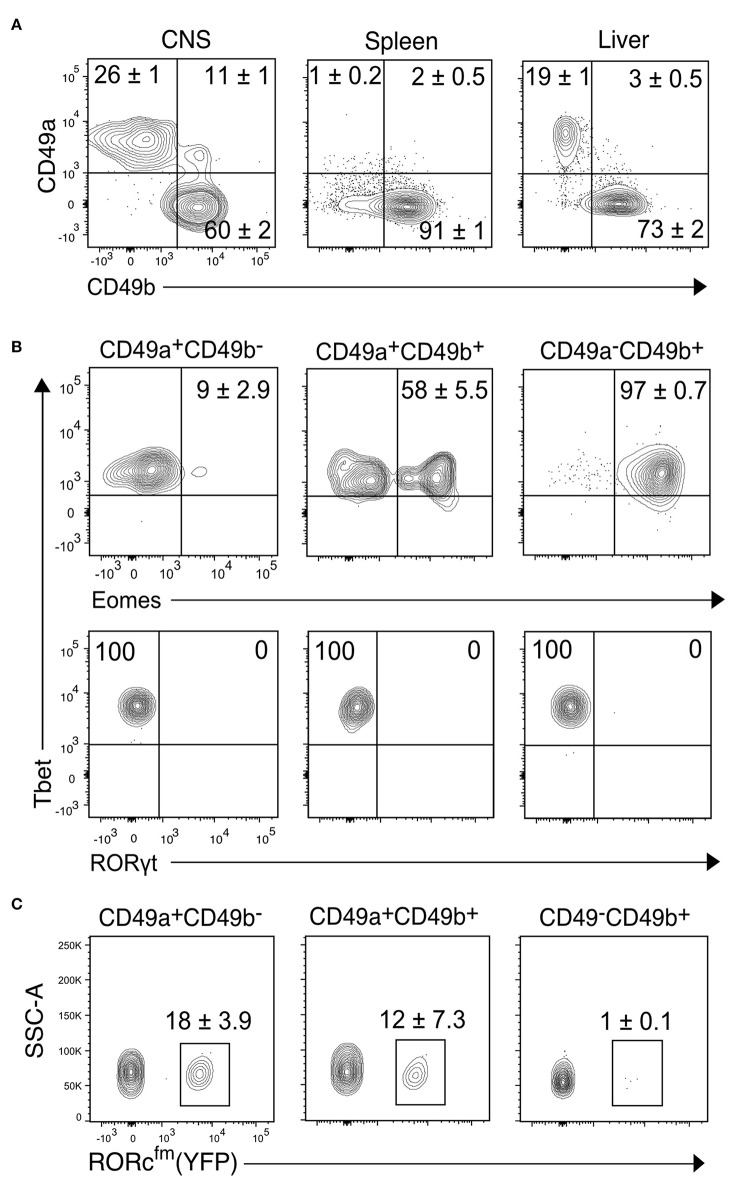
Transcription factor analysis of the NK1.1^+^ subsets of the CNS identified by CD49a and CD49b. **(A)** Representative contour plots showing the proportion of CD49a^+^CD49b^−^, CD49a^+^CD49b^+^, and CD49a^−^CD49b^+^ populations in the CD45^high^CD3^−^NK1.1^+^ cells of the CNS, spleen and liver (*n* ≥ 6, performed in three independent experiments). **(B)** Transcription factor expression analysis of the CD49a^+^CD49b^−^, CD49a^+^CD49b^+^, and CD49a^−^CD49b^+^ populations in the CD45^high^CD3^−^NK1.1^+^ cells of the CNS. Representative contour plots of the expression of Eomes, Tbet and RORγt. Numbers indicate the mean percentage ± SEM of each population (*n* = 5). **(C)** Analysis of the presence of RORc^fm+^ (YFP^+^) cells in the CD45^high^CD3^−^NK1.1^+^ cell subsets of the CNS of the RORc^cre^; Rosa26^YFP^ mice. Numbers represent the mean ± SEM percentage of YFP^+^ cells (*n* = 3).

We previously showed that migration of mature CD11b^+^ NK cells into the inflamed CNS is mediated by the fraktalkine receptor CX3CR1 (25). In this context, we also identified immature CD27^+^ NK cells in both, healthy and inflamed mouse CNS. NK cells were identified in the CNS (brain and spinal cord) from thoroughly perfused healthy C57Bl/6 mice as CD45^high^CD11b^−/low^CD3^−^NK1.1^+^(Figure 1A). This gating strategy ensured the exclusion of potentially contaminating monocytes (CD45^high^CD11b^high^), microglia (CD45^low^CD11b^high^), and NKT cells (CD3^+^NK1.1^+^) Furthermore, we confirmed that the CD45^high^CD11b^−/low^CD3^−^NK1.1^+^ population included all NKp46^+^ cells and excluded B cells, granulocytes and basophiles, which express the linage markers CD19, Gr-1, and FcεRIα, respectively (Figure 1B). To further discriminate between ILC1s and NK cells, the markers CD49a and CD49b were respectively used. Our data showed that more than 50% of the immature CD27^+^CD11b^−^ NK cells expressed CD49a and lacked the expression of CD49b. On the other hand, the mature (CD27^+^CD11b^+^) and fully mature (CD27^−^CD11b^+^) NK cells were largely CD49a^−^CD49b^+^ (Figure 2). This data suggest that a considerable fraction of the cells previously recognized as immature NK cells present in the healthy CNS might rather represent ILC1s.

### CD3^−^NK1.1^+^ Cells of the CNS Include NK Cells, ILC1s, intILC1s, and ex-ILC3s But Not ILC3s

To further characterize the CD45^high^CD3^−^NK1.1^+^ cell subsets of the CNS, we compared the proportion of the CD49a^+^ and CD49b^+^ populations to the spleen, a representative lymphoid organ that contains mostly NK cells, and the liver, a non-lymphoid organ that contains both NK and ILC1s. Similar to the liver, about 30% of CNS NK1.1^+^ cells were CD49a^+^CD49b^−^ while about 60% were CD49a^−^CD49b^+^; in addition, about 10% of the NK1.1^+^ cells expressed both CD49a and CD49b (Figure 2A). As expected, most NK1.1^+^ cells of the spleen were CD49b^+^. To confirm the identity of the identified populations in the CNS, we performed flow cytometry analysis of the transcription factors Eomes, Tbet and RORγt. The CD49a^−^CD49b^+^ subset expressed Tbet and Eomes, confirming their NK cell identity; while CD49a^+^CD49b^−^ cells were Tbet^+^Eomes^−^, indicative of an ILC1 identity. Within the CD49a^+^CD49b^+^ subset, about 50% were Eomes^+^, which could represent an intermediate stage between NK and ILC1 cells as it has been shown in the context of tumor microenvironment and in the salivary glands (26, 27) (Figure 2B). Therefore, we termed this population intermediate (int) ILC1s. None of the subsets expressed RORγt discarding the presence of NCR^+^ ILC3s within the identified populations. However, using the genetic fate mapping (fm) mice for RORγt (Rorc-Cre^Tg^; Rosa26R^YFP/+^, referred as RORc^fm^), which permanently and heritably marks all cells that expressed RORγt during their development, it was found that about a quarter of the ILC1s and intILC1s were ex-ILC3s (Figure 2C). In contrast, we found only few ILC3s in the healthy CNS compartments of adult female RORc-GFP reporter mice (Supplementary Figure 1). In sum, these results confirm the existence of different group 1 ILCs in the CNS of healthy mice that can be identified by the surface expression of the integrins CD49a and CD49b.

### CNS-ILC1s Are Distinguished From CNS-NK Cells by Their Expression of CD200R, CXCR6, TRAIL, and DNAM-1 and Higher Secretion of TNF-α

We next characterized the group 1 ILCs of the CNS, contrasting the expression of different ILC-defining markers between the ILC1s and NK cells on the healthy mouse. IntILC1s were excluded from the analysis because of their reduced number and mixed transcription factor expression.

**Figure 3:**
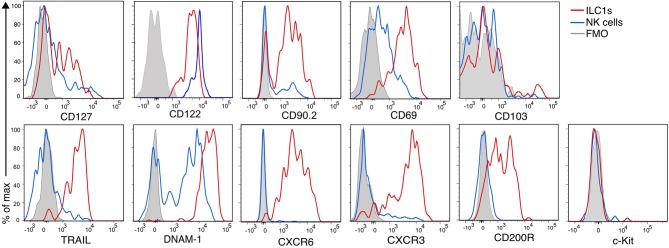
Phenotypic profile of ILC1s and NK cells of the healthy CNS. Histogram overlay of NK cells (CD49a^−^CD49b^+^) (blue line), ILC1 (CD49a^+^CD49b^−^) (red line), and FMO control (gray filled histogram) of the CD45^high^CD3^−^NK1.1^+^ populations of the CNS showing the expression of the indicated markers. Representative histograms showing concatenated samples of three mice. The expression of each marker was analyzed in at least *n* = 4 different mice performed in at least two independent experiments.

Figure 3 shows the phenotypic profile of the group 1 ILCs of the CNS. We found that few ILC1s and NK cells expressed CD127 (24 ± 2 and 7 ± 1%, respectively), but all of them expressed CD122. CD90.2, another prototypical ILC marker, was expressed in great proportion by ILC1s (81 ± 4%), nonetheless, a fraction of NK were positive for CD90.2 (39 ± 4%). The majority of ILC1s expressed the inhibitory receptor CD200R (80 ± 2%), which is also a proposed marker to distinguish NK cells from ILC1s (28). As expected, ILC1s homogeneously expressed the marker of tissue residency CD69 (79 ± 2%), but they were negative for CD103, an integrin expressed on intraepithelial ILC1s. As expected, nor ILC1s neither NK cells expressed c-Kit. ILC1s expressed perforin (95 ± 1%), but in a significant lower density than NK cells (perforin gMFI ILC1s = 2,316; NK cells = 10,561; *p* = 0.006). About half of ILC1s expressed CXCR3 (49 ± 4%), while most ILC1s homogeneously expressed TRAIL (81 ± 6%), CXCR6 (97 ± 0.8%) and DNAM-1 (96 ± 1%). NK cells did not express TRAIL, CXCR6, and CD200R. Interestingly, DNAM-1 expression on NK cells was low while all ILC1s were DNAM-1^high^. Our phenotypic profiling revealed that intILC1s share some characteristics with ILC1s like a high expression of CD69, Thy1.2, and TRAIL. However, compared to ILC1s, intILC1s had a lower expression of CD200R, CXCR6, DNAM-1, and CXCR3 (Supplementary Figure 2). In sum, the phenotypical profile of CNS CD3^−^NK1.1^+^CD49a^+^CD49b^−^ cells reveal hallmark ILC1 properties that further substantiate their identity.

### Modulation of CNS-ILC1 Chemokine Receptor Expression During Autoimmune Neuroinflammation

**Figure 4:**
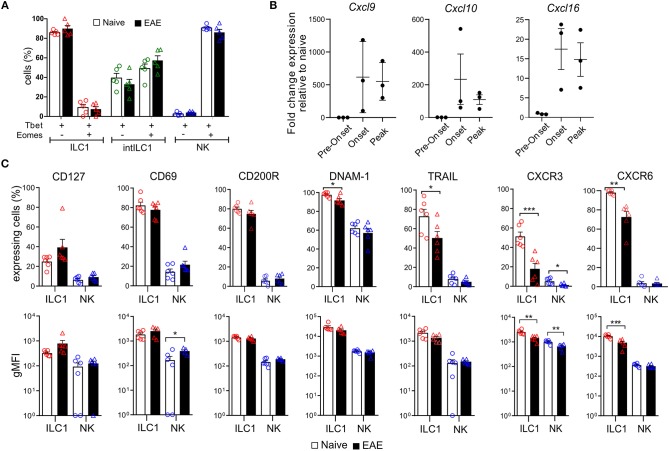
Effect of neuroinflammation on the phenotype of CNS group 1 ILCs. **(A)** Comparison of the percentage of Tbet^+^Eomes^+^ and Tbet^+^Eomes^−^ cells in the healthy (open bars) and EAE (black filled bars) mice within the ILC1 (red), intILC1 (green), and NK (blue) subsets of the CNS (*n* = 5 from two different experiments). **(B)** Quantitative RT-PCR analysis of genes encoding the ligands for CXCR3 (*Cxcl9* and C*xcl10*) and CXCR6 (*Cxcl16*); gene expression is presented as fold change relative to the expression in naive brains (*n* = 3). **(C)** Graphs showing the comparison of the percentage and gMFI of markers analyzed in Figure 3 in the healthy (open bars) and EAE (black bars) mice (*n* = 6 performed in two to three independent experiments). Data were analyzed with a *t*-test, asterisks indicate a significant difference with a *p < 0.05, **p < 0.01, or ***p < 0.001.

Next, we asked how the ILC group 1 subsets of the CNS respond to inflammation. We first analyzed whether the markers examined in Figure 3 are modulated in autoimmune CNS inflammation. For that, we compared the group 1 ILCs of the CNS of EAE mice that presented overt clinical symptoms (score 1.5–3) with healthy controls. Of note, NK cells did not acquire the expression of ILC1 markers or vice versa during inflammation and both Eomes and Tbet expression remained stable in the group 1 ILCs defined by the surface expression of CD49a and CD49b during CNS inflammation (Figure 4A), confirming the reliability and stability of those markers during autoimmune inflammation. We found that the expression of CD200R, CD69, and CD127 on ILC1s remained stable during EAE. In concordance with their activation during inflammation, there was an increase of CD69 expression in NK cells in EAE. During EAE, the proportion of ILC1s that expressed TRAIL and DNAM-1 was moderately decreased. Most striking was the decrease in the expression of the chemokine receptors CXCR3 and CXCR6 (Figure 4C), which could reflect an internalization of the receptor due to an ongoing ligand exposure within the inflamed brain. Therefore, we monitored the expression of the associated chemokines *Cxcl9, Cxcl10*, and *Cxc16* by real time qPCR in the brain of naive and EAE mice. *Cxcl9* was undetectable in naive and preonset EAE conditions and increased at onset and peak EAE. Similarly, *Cxcl10* and *Cxcl16* was detectable albeit at low levels in naive and preonset EAE brains and dramatically increased at onset and peak EAE (Figure 4B). These data strongly suggest that ILC1s are able to respond to increased chemokine gradients during CNS inflammation.

### Functional Properties of CNS-ILC1s

**Figure 5:**
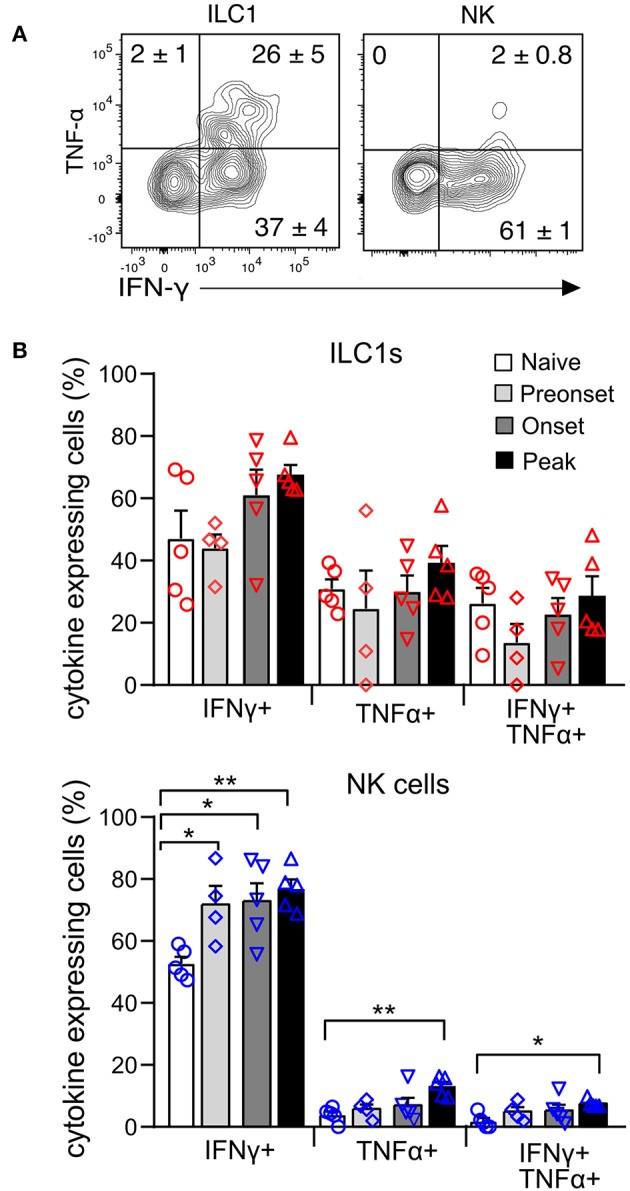
Cytokine production of CNS group 1 ILCs during EAE disease course. **(A)** Representative contour plots of the expression of IFN-γ and TNF-α in the ILC1 and NK cell subsets derived from the CNS of naive mice upon stimulation with PMA and Ionomycin. Numbers indicate the mean percentage ± SEM of each population (*n* = 5). **(B)** Graph showing the comparison of the percentage of cytokine expressing ILC1s (above) and NK cells (bottom) at different EAE stages. Differences on cytokine expression between disease stages was analyzed with a one-way ANOVA with a Bonferroni post-test (*n* = 4–5, from two different experiments), *p < 0.05, **p < 0.01.

To evaluate the functional properties of CNS-ILC1s in comparison with CNS-NK cells on the naive mouse, we investigated their ability to secrete IFN-γ and TNF-α after *ex vivo* PMA and Ionomycin stimulation. NK cells and ILC1s expressed IFN-γ in similar proportion. Notably, ILC1s secreted significantly more TNF-α than NK cells. In addition, ILC1s were also able to express both TNF-α and IFN-γ simultaneously (Figure 5A). ILC1s provide an early source of cytokines during acute infection (5, 28, 29), therefore we tested whether ILC1s were differently primed to secrete cytokines at preonset, onset and peak EAE. Interestingly, we did not observe a significant difference in the percentage of ILC1s expressing IFN-γ and TNF-α at different EAE stages upon *ex vivo* stimulation (Figure 5B). In contrast, the proportion of cytokine expressing NK cells was significantly higher as EAE disease progressed.

**Figure 6:**
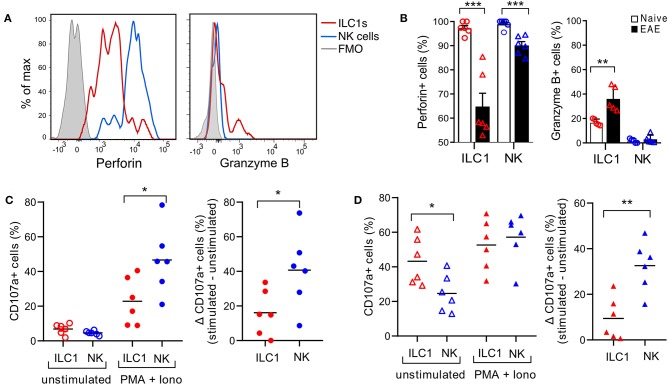
Cytotoxic potential of group 1 ILCs in the healthy and inflamed CNS. **(A)** Histograms showing the expression of intracellular cytolytic proteins perforin and granzyme B in ILC1s and NK cells present in the CNS of naive mice; representative histogram from *n* = 6 obtained in three independent experiments. **(B)** Comparison of the percentage of ILC1s and NK cells expressing perforin and granzyme B in naive and EAE mice (*n* = 5–6, three independent experiments), *t*-test. **(C)** Degranulation assay of CNS cells derived from naive mice, incubated for 4 h without stimulants or with PMA and Ionomycin in the presence of CD107a. The percentage of CD107a^+^ cells in NK cells and ILC1s is shown in the left graph. At the right, the delta of the stimulated–unstimulated condition is shown (*n* = 6 from three independent experiments). **(D)** Degranulation assay performed as **(C)** with CNS cells derived from EAE peak mice; *n* = 6 from three independent experiments; *t*-test; *p < 0.05, **p < 0.01, or ***p < 0.001.

ILC1s are generally considered poorly cytotoxic. However, we observed that in the naive CNS, all ILC1s express perforin, though in a lower density than NK cells (Figure 6A). Moreover, intracellular granzyme B was detected in a fraction of ILC1s and was absent in NK cells. Interestingly, at peak EAE we found a significant reduction of ILC1s and NK cells expressing intracellular perforin, while the frequency of ILC1s that expressed granzyme B increased (Figure 6A). Further, to evaluate degranulation potential of ILC1s, we performed a stimulation-based assay with cells derived from the CNS of naive and peak EAE mice. CD107a surface exposure was low in cells incubated in the absence of stimulants and increased upon PMA and Ionomycin stimulation in both ILC1s and NK cells of the naive CNS. However, the response to the activation was more prominent in NK cells than in ILC1s (Δ stimulated–unstimulated) (Figure 6C). Interestingly, both ILC1s and NK cells from the inflamed CNS showed a higher basal CD107a surface expression in the unstimulated condition, but again, NK cells displayed a significant increased degranulation upon stimulated compared to ILC1s (Figure 6D). These results show that ILC1s respond differently than NK cells in the CNS during autoimmune inflammation.

### Location and Dynamics of CNS Group 1 ILCs During EAE

**Figure 7:**
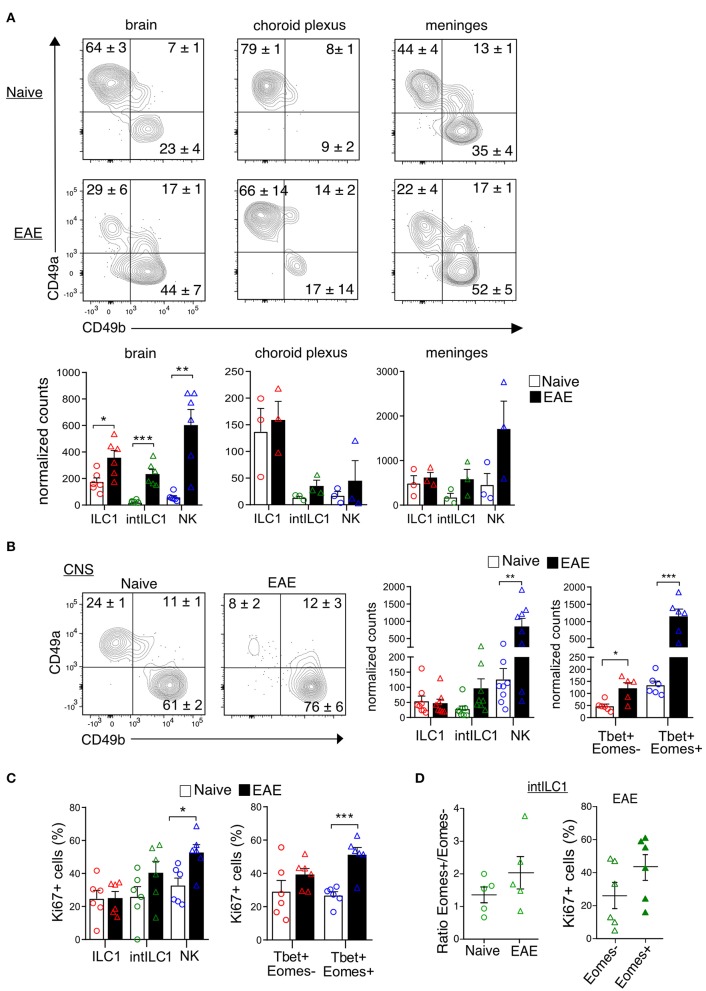
Location of group 1 ILCs within CNS compartments and their dynamics during neuroinflammation. **(A)** Representative flow cytometry plots show the percentages and standard error of each subset in the brain, choroid plexus and meninges in the naive and EAE mice. At the bottom, the graphs show the counts normalized to live single cells of each subset on each compartment. Each data point represents a single brain (*n* = 6), for the choroid plexus and meninges each data point represents pooled tissue from two mice. The difference between the naive and EAE condition was analyzed with a *t*-test; *p < 0.05, **p < 0.01, ***p < 0.001. **(B)** Representative contour plots showing the mean ± SEM proportion of group 1 ILC subsets in the whole CNS of healthy and EAE mice. At the right, the graphs show the counts normalized to the live single cell gate of each subset defined by the surface markers (left) and the transcription factors (right) (*n* ≥ 5 from at least two independent experiments). **(C)** Graphs show the proportion of Ki67^+^ cells in the group 1 ILC subsets in the healthy and EAE mice, defined by the surface markers (left) and the transcription factors (right) (*n* ≥ 5 from two different experiments). **(D)** Ratio of Eomes^+^/Eomes^−^ cells within the intILC1 population in CNS from healthy and EAE mice (right, *n* = 5, from two independent experiments). Percentage of Ki67^+^ cells in the Eomes^+^ and Eomes^−^ subset of the intILC1s of EAE mice (left, *n* = 6, from two different experiments). Data were analyzed with a *t*-test, asterisks indicate a significant difference with a *p < 0.05, **p < 0.01, or ***p < 0.001.

ILCs are enriched in barrier structures and the presence of ILCs in the meninges has been reported (30). Therefore, we next explored whether the presently described group 1 ILCs had a preferential location within the CNS compartments. We carefully removed the meninges and the CP from all the ventricles of the brain and isolated the dural meninges to separately examine the proportions of ILC1s and NK in each compartment. ILC1s, intILC1s and NK cells were present in the three compartments. Interestingly, a dominance on the proportion of ILC1s was observed in all the compartments in the naive mouse with an increase of NK cells in the EAE condition. The ILC1s were highly enriched in the CP (about 80% of the CD3^−^NK1.1^+^ gate) while only few NK cells were found there (about 10% of the CD3^−^NK1.1^+^ gate). The brain devoid of CP showed also a higher proportion of ILC1s than NK cells (64% ILC1s vs. 23% NK cells), while in the meninges, the proportion and numbers of NK cells and ILC1s were similar (44% ILC1s vs. 35% NK cells). The proportion and numbers of intILC1s were low in all the compartment of the healthy CNS. During EAE, there was an increase on the numbers of ILC1s (2-fold increase) and most notably of intILC1s (12.3-fold increase) and NK cells (10.2-fold increase) in the brain (Figure 7A). The increased numbers group 1 ILCs on the brain parenchyma during EAE could point to a proliferation of these subsets in response to neuroinflammation, therefore, we analyzed this possibility using the whole CNS. Compared to the naive mice, the proportion of ILC1s decreased (24.6 vs. 8.2%, *p* < 0.0001) while the proportion of NK cells increased (61.6 vs. 76.3%, *p* = 0.04) in the CNS during EAE (Figure 7B). When examining cell counts, it was evident that the numbers of NK cells (identified by CD49b as well by the transcription factors) increased during EAE. In line with this, the analysis of proliferation with the Ki67 marker revealed that about 50% of NK cells were actively proliferating in the CNS during EAE, while the proportion of proliferating ILC1s remained the same in the naïve and EAE mice (Figure 7C). The numbers of Tbet^+^Eomes^−^ cells also increased during EAE (Figure 7B), however, a significant increase of proliferating cells was not detected in the Tbet^+^Eomes^−^ cells nor in the intILC1s or ILC1s (Figure 7C). As the Tbet^+^Eomes^+^ cells were the most proliferative during EAE, we also analyzed whether within the intILC1s the Eomes^+^ subset proliferated more than the Eomes^−^ subset. We did not detect an increase on the proportion of Eomes^+^ intILC1s nor a higher proportion of Ki67^+^ cells in the Eomes^+^ intILC1s during EAE (Figure 7D). These data indicate that NK cells, but not ILC1s nor intILC1s, proliferate readily in the inflamed CNS and that the increased numbers of intILC1s and ILC1s in the brain parenchyma during inflammation might derive from an infiltration of cells from other CNS compartments.

## Discussion

In this study, we characterized the ILC group 1 subsets of the CNS and investigated their response to local autoimmune inflammation using the EAE model of MS. We determined that the CD45^high^CD3^−^NK1.1^+^ cells present in the CNS include three different populations that can be defined by the expression of the integrins CD49a and CD49b. We confirmed that these populations are distinct based on the expression of the transcription factors Eomes and Tbet (Figure 2B), which control the transcriptional programs of NK cells and ILC1s, respectively (31, 32). In addition, the analysis of the expression of signature proteins of NK cell and ILC1s substantiate their differential identity (Figure 3).

In our previous studies, we observed that phenotypically mature and immature NK cells were present in the CNS during steady state in mice. CD27 is also expressed in ILC1s, and a closer look revealed that indeed many of the cells recognized as immature CD27^+^ NK cells were rather ILC1s (Figure 1C). A mass cytometry characterization of the brain's immune compartment also identified the presence of CD45^high^NK1.1^+^CD49b^+^ NK cells (33), the authors showed that these cells express CD27 and IL-2R. In concordance, we found that both NK cells and ILC1s express CD122, which conforms the beta subunit of the IL-2 and IL-15 receptors. We further demonstrate that CD45^high^NK1.1^+^CD49b^+^ cells in the CNS have a predominant CD11b^+^ expression, while this marker is not expressed in the ILC1s (CD45^high^NK1.1^+^CD49a^+^CD49b^−^) cells, coinciding with the known gene signature of NK cells and ILC1s (34).

In addition to ILC1s we identified in the CNS a small (10%) but stable population of CD49a^+^CD49b^+^ intILC1s, comparable to the intermediary population described by Gao et al. in the tumor microenvironment (26). Interestingly, within this population, about 50% were Eomes^+^. This proportion remained stable during neuroinflammation (Figures 4A, 7D) and differ from ILC1s in the expression of CXCR6 and CD200R (Figure 3 and Supplementary Figure 2).

Furthermore, we found that a fraction of the ILC1s in the CNS have an ILC3 origin as our experiments with the RORc^fm^ mice revealed (Figure 2C). It is therefore possible that in most tissues, the cells recognized as ILC1s contain a fraction of ex-ILC3, as was recently shown in the uterine ILC1s (35). In line with this, we also found a similar percentage of ex-ILC3s in the ILC1 subset of the liver (Supplementary Figure 1A). On the other hand, we found very few ILC3s (about 2% of Lin^−^ cells) in the healthy CNS compartments of adult mice (Supplementary Figure 1B), suggesting that the ILC3 source of ex-ILC3 cells found in the CNS originates during ontogeny. It was shown in adoptive transfer experiments that RORc^fm+^ ILCs adapt their phenotype to the tissues they invade (36), for this reason we included in our analysis the ex-ILC3s that are part of the ILC1s. On a functional level, ex-ILC3s were found to exacerbate experimental colitis (37). In addition, it was shown that RORc^fm+^ ILCs in the spleen have a more potent anti-tumoral properties than the RORc^fm−^ ILCs (36). It is therefore crucial to study the plasticity and the specific contribution of the ILC1, ILC3, and ex-ILC3 subsets in CNS pathology. Questions we are currently addressing in the laboratory.

Our phenotypic profile revealed that CNS-ILC1s express hallmark markers of ILC1s in other organs, such as TRAIL, CXCR6, CXCR3, DNAM-1, and CD200R. However, we found that few CNS-ILC1s expressed IL-7Rα (about 30%), which remained stable during neuroinflammation (Figures 3A, 4A) The expression of CD127 is a defining marker of helper ILCs (3). However, in the case of group 1 ILCs, IL-15 but not IL-7 is required for their differentiation and maintenance (5). In concordance, ILC1s in the liver, skin and uterus present moderate or no expression of IL-7Rα (38).

ILC1s expressed the marker of tissue residency CD69, but they did not express CD103, an integrin expressed in intraepithelial ILC1s (39). In concordance with a recent report (28), we found expression of CD200R in most ILC1s (80 ± 2%), while NK cells did not express this marker. Notably, CD200R expression remained stable on ILC1s during neuroinflammation, making it a suitable candidate to identify them under different inflammatory conditions. The ligand CD200 is expressed by endothelial cells and neurons (40) and the blockage of the CD200R-CD200 axis led to an aggravation of EAE disease (41), while an improved outcome was observed when CD200R activation was enhanced (42). Thus, CD200R^+^ ILC1s could also play a role in mediating anti-inflammatory effects during neuroinflammation.

CNS-ILC1s expressed the chemokine receptors CXCR3 and CXCR6 (Figure 3A). CXCR3 was also expressed in a fraction of immature NK cells but CXCR6 was totally absent in NK cells. CCR2, CCR6, and CCR7 expression was also absent from both ILC1s and NK cells of the CNS (not shown). Interestingly, both CXCR3 and CXCR6 became downregulated during EAE (Figure 4A). G protein-coupled receptors can be rapidly internalized and degraded after ligand activation. In this line, it was shown that CXCR3 is internalized in activated T cells after engagement with their ligands (43). Consistent with previous reports (44, 45), we found an increase in the expression of *Cxcl9* and *Cxcl10* at onset and peak EAE (Figure 4B). CXCL16 expression has been identified in endothelial cells of the CNS, astrocytes, microglia, and neurons (46, 47) and its expression has been shown to be upregulated *in vitro* by IFN-γ and TNF-α (46). Furthermore, an elevated concentration of CXCL16 has been found in the CSF of MS patients (48). In line with this, our RT-qPCR analysis revealed a low expression of *Cxcl16* in the brain of the naive mice (not shown) and an increase at onset and peak EAE (Figure 4B). Together, these data suggest that the decrease of CXCR3 and CXCR6 on ILC1s during EAE may reflect an ongoing and functional activation of these receptors. The CXCL16-CXCR6 axis has been shown to promote neuroprotection in a model of glutamate excitotoxicity and ischemia (47, 49). Furthermore, CXCR6^+^ ILC1s have been assigned a memory function in the liver and uterus (35, 50). Altogether, these data suggest that CNS CXCR6^+^CXCR3^+^ ILC1s are able to home to different locations inside the CNS, where they could mediate neuroprotective and memory functions.

In general, helper ILC1s are known to lack cytolitic activity, however, a cytotoxic potential of Eomes^−^ “tissue resident NK cells” in the context of cancer has been observed [reviewed in (51)]. We showed that CNS resident ILC1s cells are positive for perforin, but express it in a lower level compared to NK cells (Figure 6A). In addition, similar to liver and salivary gland ILCs (27, 52), CNS resident ILC1s express the apoptosis-inducing ligand TRAIL. Interestingly, we found a slight decrease in TRAIL expression during neuroinflammation (Figure 4C). TRAIL can be released in a soluble form (53, 54) and it was shown that their receptors are endocytosed together with bound TRAIL to regulate the apoptotic signaling (55). Therefore, the decrease in TRAIL^+^ ILC1s could reflect either a cleavage to generate soluble TRAIL, or an internalization after being bound to the death receptors in target cells. Although neurons expressing TRAIL-R2 are susceptible to TRAIL-mediated killing by encephalitogenic T cells (56), it has also been shown that TRAIL has immunomodulatory effects in EAE (57, 58). A proposed mechanism involve the lysis of autoreactive T cells (59). In the same line, DNAM-1 has been implicated in the interaction of NK cells with T cells via its ligand CD155, which mediates NK cell cytotoxicity against CD4^+^ T cells (19). Notably, we found that all CNS-ILC1s are DNAM-1^high^, and that some of them loss their expression during neuroinflammation (Figure 4C). Based in this observations we further explored the degranulation potential of ILC1s in the context of neuroinflammation. Strikingly, we found that both ILC1s and NK cells contain less intracellular perforin during EAE (Figure 6B). At the same time, they displayed high degranulation as shown by an increased CD107a surface expression even in the unstimulated conditions (Figure 6D). Therefore, the decrease in intracellular perforin could indicate its release by active degranulation during neuroinflammation. Furthermore, ILC1s but not NK cells expressed granzyme B, which increased in EAE. Different kinetics of granzyme B and perforin *de novo* synthesis could explain why granzyme B increases while perforin decreases in ILC1s during EAE. Overall, our data indicate that ILC1s of the CNS are capable of degranulation, which, in comparison to NK cells, is however lower upon PMA and Ionomycin stimulation (Figures 6C,D). Taken together, our data suggest a potential role of CNS-ILC1s to limit inflammation by interacting and lysing autoreactive T cells, a hypothesis currently being tested in our laboratory.

It has been shown that ILC1s produce larger amounts of TNF-α and other cytokines than NK cells (6, 38). In concordance, we found that ILC1s stimulated *ex vivo* produced significantly more TNF-α than NK cells (Figure 5A). However, contrary to models of acute infection (5, 28, 29, 60), we did not observed a higher production of cytokines in ILC1s at early stages of EAE disease (Figure 5B).

Few studies have examined the role of ILCs in EAE. Using an antibody-mediated depleting strategy of Thy1^+^ ILCs, Mair and Becher (61) showed that disease course was not affected by the absence of ILCs. Although their focus was on Thy1^+^Sca^+^IL7-Rα^+^ ILCs, their depleting strategy presumably affected all ILC subsets including the presently described ILC1s. ILC3s were shown to accumulate in the meninges during EAE (62). More recently, Kwong et al. showed that in the Th17 adoptive transfer EAE model, meningeal Tbet-dependent NKp46^+^ ILCs contribute to the inflammatory microenvironment that promotes Th17 cell maintenance and migration to the parenchyma (30). We found that ILC1s reside not only in the meninges, but that they are specially enriched in the CP (Figure 7A). The CP appears to be involved in the initiation of the inflammation in EAE (63) and has been proposed as a key entry gate to the CNS, which is tightly regulated by a balance of type I and type II interferons (64). IFN-γ-producing T cells residing in the CP were shown to mediate licensing and gatekeeper functions during neuroinflammation and aging (65, 66). Similarly, we anticipate that ILC1s residing in the CP could exert a gatekeeper function, as it was also shown for the ILCs residing in the meninges (30).

Finally, we found that the inflammatory microenvironment of the MOG-induced active EAE did not induce the proliferation of ILC1s but favored the proliferation of NK cells as shown by the increased expression of Ki67 (Figure 7C). Nevertheless, the number of ILC1s and especially of intILC1s was increased in the brain parenchyma during EAE (Figure 7A). The increase in the numbers of intILC1s and ILC1s could therefore derive from a cell infiltration from the meningeal or CP compartment, a hypothesis that is supported by the upregulation of *Cxcl9, Cxcl10*, and *Cxcl16* shown in the inflamed brain parenchyma. A NK cells recruitment from the periphery into the CNS during inflammation has been previously shown (25, 67, 68), we additionally demonstrate that NK cells are able to proliferate in the CNS contributing to the increased NK cell numbers observed in the EAE peak disease.

In sum, our data portray for the first time the characteristics of group 1 ILCs in the CNS. The presented results strongly hint into an immunomodulatory function of CNS-ILC1s. Further studies should shed light into the mechanisms in which group 1 ILCs mediate CNS homeostasis and pathology.

## Data Availability Statement

The datasets generated for this study are available on request to the corresponding author.

## Ethics Statement

The animal study was reviewed and approved by Landesamt für Gesundheit und Soziales.

## Author Contributions

SR-S designed and performed the experiments, analyzed and interpreted the data, and wrote the manuscript. AD, RB, CS, CF, DB-S, and LH assisted in the experiments. ID edited the manuscript and provided technical advice on meningeal preparation and staining. CR helped with the study design, provided the technical advice on ILC staining, and edited the manuscript. CI-D conceptualized and supervised the study, revised and edited the manuscript.

### Conflict of Interest

The authors declare that the research was conducted in the absence of any commercial or financial relationships that could be construed as a potential conflict of interest.
