# Supplementary material for: The Central Nervous System Contains ILC1s That Differ From NK Cells in the Response to Inflammation
Source: Front Immunol. 2019 Oct 10;10:2337. doi: 10.3389/fimmu.2019.02337 (PMC6795712; doi:10.3389/fimmu.2019.02337)
Supplement: Supplementary file 1 [file Data_Sheet_1.docx]

Supplementary Figures


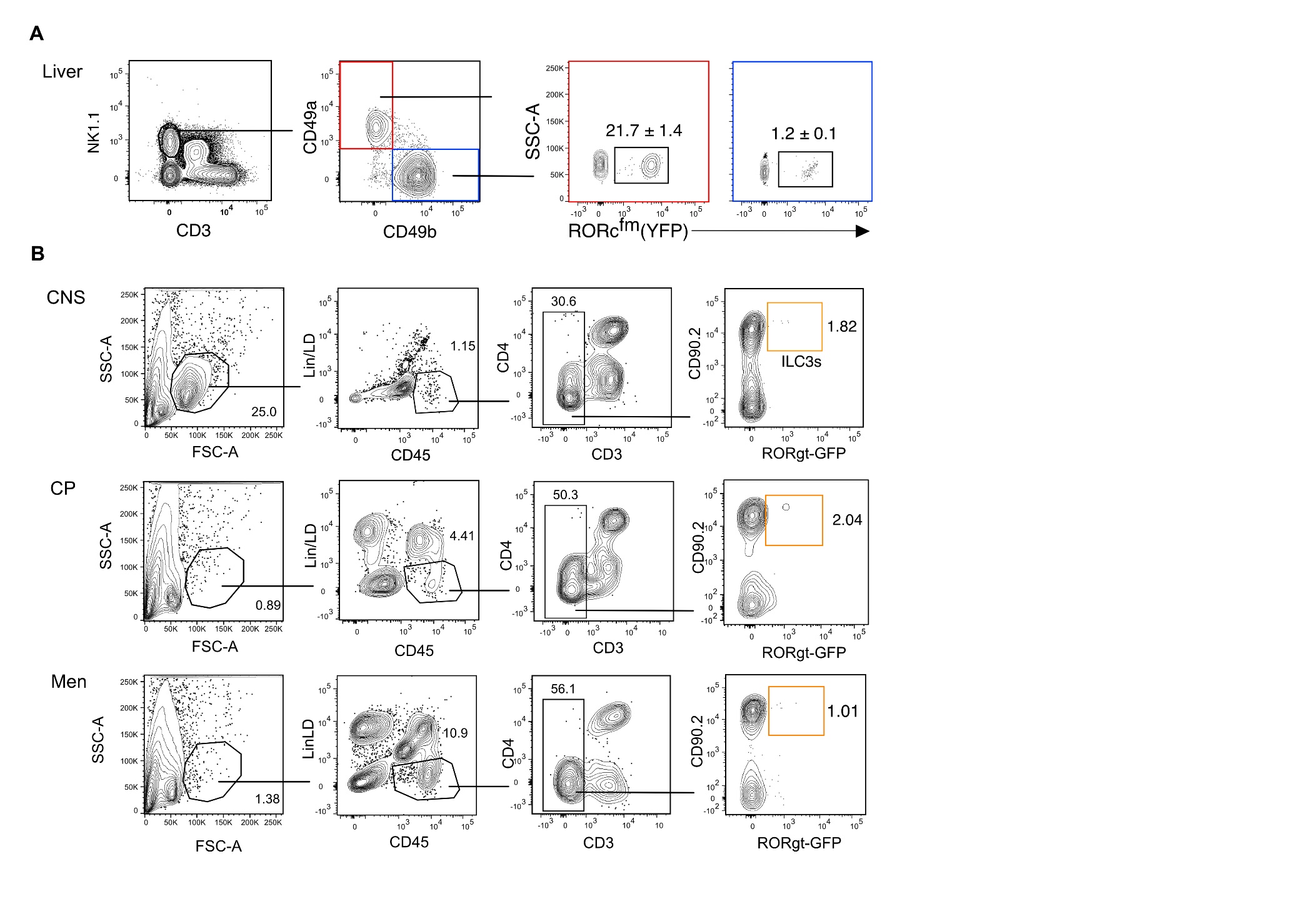


**Supplementary Figure 1. RORc^fm+^ expression in liver ILC1s and examination of ILC3s in the CNS compartments using the RORgt reporter mouse (RORgt-GFP).** (A) Contour plots show the expression of YFP in ILC1s (CD3^+^NK1.1^+^CD49a^+^CD49b^-^) and NK cells (CD3^-^NK1.1^+^CD49a^-^CD49b^-^) of the liver of RORc-fate mapping mice. Numbers represent the mean percentage of 3 mice. (B) Contour plot showing the gating strategy to identify ILC3s in the CNS (brain + spinal cord), choroid plexus (CP) and meninges (men) of healthy RORgt-GFP mice. ILC3s were identified as Lin^-^ (B220, CD11c, Ly6G/D, F4/80, FcεR1α) CD45^+^CD3^-^CD4^-^CD90.2^+^GFP^+^ cells. Representative plots of 2 mice, pooled CP and meninges.


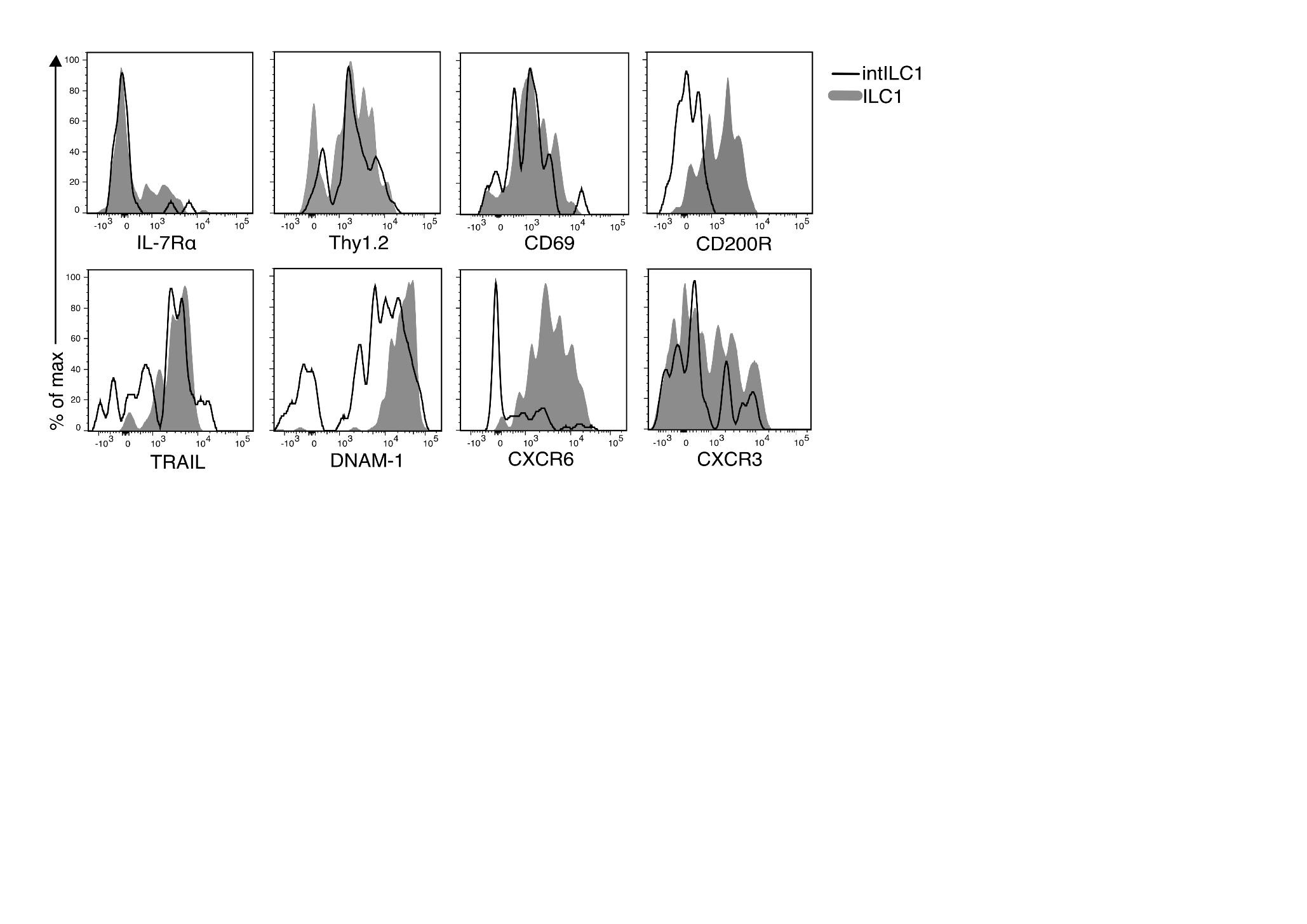


**Supplementary Figure 2. Phenotypic profile of intILC1s of the healthy CNS.** Histogram overlay of intILC1s and ILC1s showing the expression of the indicated markers. Representative histograms showing concatenated samples of 3 mice. The expression of each marker was analyzed in at least 4 different mice performed in at least 2 independent experiments.
